# Mapping the supportive care needs and quality of life of adult survivors of childhood cancer at a comprehensive cancer center in the Middle East

**DOI:** 10.1038/s41598-024-60567-9

**Published:** 2024-05-29

**Authors:** Nedal Al-Rawashdeh, Rana Damsees, Haneen Abaza, Khawlah Ammar, Ibrahim Alananzeh, Amal Abu Ghosh, Shireen Al-Awady, Hashem Abu Serhan, Khaled Al-Jafari, Omar Awadallah, Zaid Al-Jafari, Leen Abu Serhan, Sarah Irshaidat, Emad Obeidat, Amal Al-Omari

**Affiliations:** 1 The Office of Scientific Affairs and Research, King Hussein Cancer Center, Amman, 11941 Jordan; 2 Clinical Trials Unit, Sheikh Shakhbout Medical City, Abu Dhabi, UAE; 3 Department of Science, Technology and Research, Ministry of Education, Abu Dhabi, UAE; 4 School of Nursing, Faculty of Science, Medicine and Health, University of Wollongong, Northfields Avenue, Wollongong, NSW 2522 Australia; 5 Departments of Pediatrics, King Hussein Cancer Center, Amman, 11941 Jordan

**Keywords:** Cancer survivors, Supportive care, Needs assessment, Quality of life, Childhood, Cancer, Health care

## Abstract

Assessing unmet needs is crucial to achieving quality care and patient satisfaction. Between September and December 2021, we assessed unmet supportive care needs in a consecutive sample of adult survivors of childhood cancer at KHCC (King Hussien Cancer Center). Two hundred and ninety-seven adult survivors of childhood cancer completed the study questionnaire. The average needs score across all domains was 24.80 (SD = 19.65), with the financial domain scoring the highest 30.39 (SD = 31.95) and sexuality scoring the lowest 7.67 (SD = 19.67). Using a multivariate linear regression model, female gender was independently associated with significantly high scores in all need domains (p < 0.001), except for sexuality. Monthly income, comorbidities, socioeconomic challenges, time since diagnosis, and age at diagnosis have emerged as predictors of needs in many domains. Mean quality of life (QoL) was significantly and inversely associated with the mean score in multiple domains: psychological (p** < **0.001), sexuality (p = 0.038), financial (p** < **0.001), and overall needs (p = 0.004). Following a content analysis of qualitative data, educational difficulties, and work-related challenges were identified as other unmet needs. Cancer experiences during childhood significantly influence supportive care needs in adulthood. There is a need for more tailored studies assessing different populations of cancer survivors and avoiding the one-size-fits-all survivorship care.

## Introduction

Childhood cancer is a broad category of rare cancers classified according to age^1^. The most common types are leukemia, CNS tumors, and lymphomas. Because of advanced diagnostic techniques, surgical and radiation therapy advancements, combination chemotherapy, and robust support care, survival rates for most childhood cancers have increased significantly over the last few decades, with disparities between developing and developed countries. In developed countries, the average survival rate exceeds 80%, in comparison with low and middle-income countries where the same rate is approximately 20%^2–5^. This dramatic increase in survival rates has resulted in a new and growing population of long-term childhood cancer survivors, which did not exist a few decades ago. It is currently estimated that one in every 640 young adults aged 20 to 39 is a survivor of childhood cancer^5^.

Cancer is a chronic disease that can cause lifelong physical and psychological late effects after diagnosis or treatment^6^. Unfortunately, significant long-term morbidity and mortality are associated with childhood cancer treatment, and the incidence continues to rise long after treatment completion. As a result, cancer survivors face a variety of short- and long-term physical and psychosocial consequences, such as persistent fatigue, pain, musculoskeletal problems, peripheral neurological symptoms (e.g. polyneuropathy), body image issues, and sexual dysfunction, amongst other issues. These conditions can range from mild to severe, temporary to lifelong, and significantly impact survivors' quality of life^7^. Furthermore, cancer survivors face socioeconomic challenges such as returning to work, loss of income, and difficulties obtaining insurance, financial loans, and Mortgages^8^.

The physical and psychological consequences of cancer and treatment are well addressed in the literature. According to Meeske et al. education, employment, insurance, military service, marriage, child-bearing decisions, recreation, and value formation are all lifestyle issues that may be impacted by cancer survivorship^9^. Comprehensive cancer care entails not only curative efforts but also consideration of a wide range of patient needs; including needs assessment tools to quantify the gap between the patients experience of service and the actual service provided in order to highlight deficiencies in service delivery^10^. By meeting these needs, we can improve the overall quality of life (QoL) for patients, and enhance their cancer care experience.

There have been a number of findings from the childhood cancer survivor study (CCSS) that collectively shed light on the multifaceted challenges faced by survivors of childhood cancer. Key among these are the long-term risks of developing second neoplasms^11^. Additionally, survivors often face physical performance limitations, indicating the lasting impact of cancer and its treatment on physical abilities^12^. Psychological challenges also emerge as a significant concern, underscoring the importance of mental health support^13^. Moreover, the adverse effects stemming from long-term radiation exposure^14^.

In Jordan, studies that highlight the needs of childhood cancer survivors are deficient. Two studies conducted by Alananzeh et al. revealed that the needs of adult cancer survivors were mainly related to physical and psychological aspects^15,16^. According to Hendriks et al. childhood cancer survivors have several unmet needs after treatment completion. Survivors reported a lack of psychosocial support, as well as a strong need for personalized, interdisciplinary, evidence-based long-term follow-up care, including adequate psychosocial services for all childhood cancer survivors^17^.

We recognize that knowledge gaps regarding supportive care needs exist in Jordan and the surrounding region, and assessing them is critical to providing quality care and achieving patient satisfaction. Assessing these needs is crucial for delivering quality care and ensuring patient satisfaction. While our previous research has shed light on the needs assessment of adult cancer survivors, along with predictors of their needs and their association with Quality of Life (QoL)^18^, the current study aims to fill a critical gap by specifically addressing the unique supportive care needs of Jordanian survivors. The importance of this study comes from the fact that it focuses on a population that has been underrepresented in cancer survivorship research “adult survivors of childhood cancer” in Jordan. The distinct needs of Jordanian survivors may differ from those in other regions due to cultural, social, and healthcare system differences. For instance, the way cancer and its aftermath are perceived and managed in Jordanian society, the availability and accessibility of healthcare resources, and specific challenges faced by survivors in this region, could significantly influence their supportive care needs. By identifying these unique needs, our study not only contributes to the global understanding of cancer survivorship but also paves the way for developing tailored interventions and policies that are culturally sensitive and effective in the context of Jordan and similar Middle Eastern countries.

This study continues on a series of studies conducted at King Hussein Cancer Center (KHCC), focusing on the unmet supportive care needs of cancer survivors. In this paper, we report the supportive care needs of adult survivors of childhood cancers, and their association with QoL, to better understand unmet needs and propose an improved survivorship care plan.

## Materials and methods

### Design

This cross-sectional study was conducted between September 2021 and December 2021. A consecutive sample of adult survivors of childhood cancer was recruited from KHCCs pediatric survivorship clinic. We used a modified version of the Supportive Care Needs Survey 34 item Short Form (SCNS-SF34) which assesses survivors needs across five domains (psychosocial, health systems/information, physical/daily living, patient care/support, and sexuality)^19^. Eligible patients were adults diagnosed with cancer at age < 18 years, off-treatment, free of cancer for at least two years, and seen at the KHCCs survivorship clinic. Participants were also asked open-ended questions at the end of the survey to identify other needs and barriers to their survivorship care.

### Study setting

This study was carried out at the King Hussein Cancer Center (KHCC) located in Amman, Jordan. KHCC is the only hospital in Jordan that offers all forms of cancer care and follows a collaborative approach in cancer management. KHCC has been recognized with multiple international accreditations that serve as evidence of the excellent standard of care provided by the center. Each year, the center delivers comprehensive cancer treatments for more than 5000 new cases. The majority of the patients (around two-thirds) are Jordanian nationals, and the remainder are from other Arab countries.

### Participants

Childhood cancer survivors were identified through the KHCC cancer registry. Survivors of pediatric cancer are referred to a pediatric survivorship clinic once they have completed one year since the end of therapy and had no evidence of disease recurrence. Participants were recruited from the pediatric survivorship clinic and were eligible if they were ≥ 18 years at the time of study entry, diagnosed with cancer at age ≤ 18 years, off-treatment, and deemed cancer-free for at least two years. Participants were invited to take part in this study during the period from September 2021 to December 2021 via a phone-based interview due to the COVID-19 restrictions. Before taking part in the study, verbal consent from the patients was obtained over the phone. Then they were asked to provide socio-demographic and clinical information, as well as information regarding the financial implications of their cancer diagnosis. Following this, they were introduced to a questionnaire consisting of 45 questions related to their supportive care needs and overall quality of life, in addition to open-ended questions regarding any further needs or barriers to survivorship care. This study was conducted according to the guidelines of the Declaration of Helsinki and approved by the Institutional Review Board of King Hussein Cancer Center (Study Number: 21 KHCC 21F). Informed consent was obtained verbally from all subjects involved in the study. All study activities were performed in accordance with the Declaration of Helsinki.

### Survey questionnaire

The study employed several measures, including the SCNS-SF34 short-form questionnaire, which is a validated self-report questionnaire consisting of 34 items. It is designed to evaluate cancer patients’ perceived needs in five areas: psychological (10 items), health system and information (11 items), physical and daily living (5 items), patient care and support (5 items), and sexuality (3 items)^18–20^. Participants were asked to indicate the level of need required for each item on the questionnaire over the previous six months using a 5-point Likert scale. A score of 1 corresponded to no need (not applicable) or no issue, a score of 2 indicated no need (satisfied), and scores of 3, 4, and 5 indicated low, moderate, and high levels of unmet need, respectively. Additional 11 questions were added to the questionnaire. Of these, six were adapted from the SCNS-LF59, and the remaining five were suggested by the study team to cover fertility and financial needs based on their experience in treating cancer patients at KHCC. In addition, the patients were asked to rate their overall quality of life using the global health status/QoL scale, which ranged from 1 (very bad) to 7 (excellent). The original Supportive Care Needs Survey Short Form (SCNS-SF34) and selected elements from its comprehensive counterpart, the Long Form (SCNS-LF59), were used in this study without change. Both instruments are widely acclaimed and have undergone extensive validation, demonstrating consistent reliability in assessing the supportive care needs of cancer patients^20–23^. The validity and reliability testing was performed on a small pilot sample and confirmed for the whole study sample in the previous study on adult cancer survivors^18^. Participants were also invited to articulate any unaddressed needs beyond the scope of the questionnaire, ensuring a comprehensive assessment of their unique supportive care requirements^24,25^. The validity and reliability of a single-item measure of quality of life, such as the global health status/QoL scale, have been explored and established previously. A study by Yohannes et al. focused the psychometric properties of the single-item measure, highlighting its strong correlation with other established quality of life measures. The sensitivity and specificity of the single-item global quality of life scale were explored, establishing its predictive ability for health-related quality of life scores. The research found that the single-item scale had a good balance of sensitivity and specificity, indicating its effectiveness in predicting quality of life outcomes^26^.

### Statistical analysis

Frequencies and percentages were used to present categorical data, while means and standard deviations were used for continuous variables. Bivariate analysis was performed to test the relationship between independent variables and supportive care needs domains. This was done by comparing means between different groups using an independent sample t-test and one-way ANOVA, followed by a Bonferroni post-hoc test. Pearson’s correlation was used to determine the correlation between the mean overall quality of life score and the mean scores in all domains. Participant characteristics were also tested for their association with mean quality of life scores using independent sample t-tests and one-way ANOVA. Finally, a multivariate generalized linear model was used to identify predictors of supportive care needs in each domain.

To standardize the raw need scores, the number of items in each domain was taken into account. For a given domain with M questions and a maximum value of k for each item (in this study, k = 5), the standardized score was calculated using the formula: (*total raw score − M*)100*/*[*M* (*k − *1)]. This resulted in a score ranging from 0 to 100 for each domain, where a higher score indicates a greater perceived need for support in that domain^19,20^. Additionally, a linear transformation was applied to standardize the overall quality of life (QoL) raw score, which was calculated using the formula: score = (raw score–1)/range 100, where the range is the difference between the maximum and minimum possible values of the raw score. This transformation also resulted in a score range from 0 to 100^27^.

The study addressed other needs by utilizing open-ended questions. The qualitative component of the study sought to complement the structured data obtained from the standardized questionnaires by exploring the breadth of survivors' experiences in their own words. This approach allows for the emergence of themes that may not be encapsulated by pre-defined survey items, providing a richer and more nuanced understanding of survivors' needs. Content analysis was employed as the methodological approach for examining these responses due to its systematic and objective framework for quantifying and analyzing the presence of certain words, themes, or concepts within qualitative data. This method is particularly well-suited to identifying and enumerating the most frequently mentioned unmet needs, as it allows for the quantification of data that can highlight prevalent themes across a large sample. This approach not only facilitated a rigorous examination of the data, ensuring unbiased results, but also proved adaptable to the study's cultural context^28,29^.

Statistical analyses were conducted using SPSS for Windows, version 28.0 (SPSS Inc., Chicago, IL, USA), and two-sided p-values were calculated. A p-value of ≤ 0*.*05 was considered statistically significant.

## Results

### Study sample

Six hundred and twenty-eight eligible patients were identified; 27 of them refused to participate, 44 did not pick up after dialing their number three times, while the remaining eligible patients could not be contacted due to disconnected phone numbers. Two hundred and ninety-seven adult survivors of childhood cancer consented to participate and completed the study questionnaire. Their mean age was 22.39 (SD = 3.5) years, and 59.3% were males. Most of the participants were diagnosed during their early or late adolescence age (54.9%) and hematological malignancies were the most common diagnoses (65.3%). The vast majority of participants were unmarried (82.2%) and living with their parents (81.8%). Additionally, most of the participants were unemployed (64.3%) and living in the capital Amman (59.9%). Other descriptive characteristics are listed in Table 1.

**Table 1:** Characteristics of study participants, n = 297.

Characteristic	Level	n (%)
Gender	Male	176 (59.3)
	Female	121 (40.7)
Age at diagnosis	≤ 5 toddler early childhood	60 (20.2)
	6–11 middle childhood	74 (24.9)
	≥ 12 early late adolescence	163 (54.9)
Current age	≤ 22 years	167 (56.2)
	> 22 years	130 (43.8)
Living situation	Alone	6 (2.0)
	With a partner	48 (16.2)
	With parents	243 (81.8)
Socioeconomic challenges	Yes	132 (44.4)
	No	165 (55.6)
Monthly income	None	54 (18.2)
	≤ 500 JD	184 (62.0)
	> 500 JD	59 (19.9)
Marital status	Not married	244 (82.2)
	Married	53 (17.8)
Employment	None	191 (64.3)
	Full-time	80 (26.9)
	Part-time	26 (8.8)
Level of education	Less than high school	53 (17.8)
	High school (Tawjihi)	78 (26.3)
	Diploma	24 (8.1)
	Bachelor	139 (46.8)
	Graduate studies	3 (1.0)
Governorate	Amman	178 (59.9)
	Zarqa	47 (15.8)
	Balqa	19 (6.4)
	Irbid	18 (6.1)
	Others (8 cities)	35 (11.8)
Time since diagnosis	≤ 5 years	41 (13.8)
	6–10 years	110 (37.0)
	> 10 years	146 (49.2)
Type of cancer	Hematological	194 (65.3)
	Solid tumors	75 (25.3)
	Brain cancer	28 (9.4)
Cancer stage	I	5 (1.7)
	II	23 (7.7)
	III	17 (5.7)
	IV	8 (2.7)
	N.A	244 (82.2)

*JD* Jordanian dinar. 500 JD ≈ 700 US Dollar.

### Unmet supportive care needs

**Figure 1:**
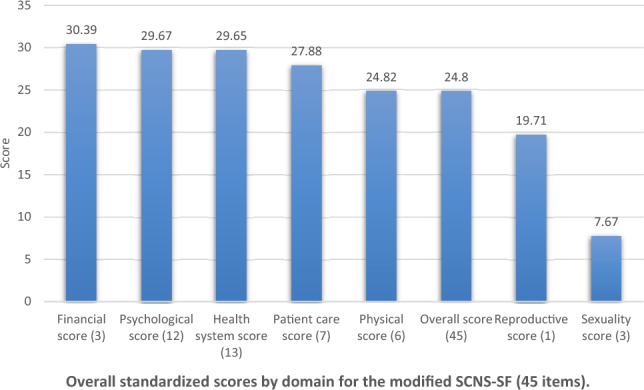
Overall standardized scores by domain for the modified SCNS-SF (45 items).

The supportive care needs in our study were quantified using a scoring system with a range from 0 to 100 across all domains, where higher scores indicate greater unmet supportive needs. The overall needs score was calculated as an average across all domains, providing a comprehensive measure of unmet needs. The average overall needs score across all domains was 24.80 (SD = 19.65), with the financial domain scoring the highest 30.39 (SD = 31.95), and sexuality scoring the lowest 7.67 (SD = 19.67), mean scores for other domains are listed in Fig. 1, the score ranges are from 0 to 100 in all domains.

The highest unmet supportive care needs that were reported by cancer survivors of childhood cancers were Money to cover living expenses (54.8%) under the financial domain, followed by Fears about cancer returning (42.4%) under the psychological domain, and To have one member of hospital staff with whom you can talk to about all aspects of your condition, treatment and follow-up (38.2%) under health system domain. The lowest unmet supportive care need was Changes in your sexual relationships (5.2%) under the sexuality domain. Other needs are listed in Table 2.

**Table 2:** Prevalence of supportive care needs among study participants; all questions, 45 items (no need vs. some need).

Domain	Supportive care needs	No need	Some need
		n (%)	n (%)
Physical	Lack of energy and tiredness	185 (62.3)	112 (37.7)
Physical	Pain	191 (64.3)	106 (35.7)
Physical	Feeling unwell a lot of the time	195 (65.7)	101 (34.0)
Physical	Not sleeping well	221 (74.7)	76 (25.6)
Physical	Work around the home	221 (74.4)	74 (24.9)
Physical	Not being able to do the things you used to do	229 (77.1)	68 (22.9)
Psychological	Fears about cancer returning	171 (57.6)	126 (42.4)
Psychological	Uncertainty about the future	176 (59.5)	120 (40.5)
Psychological	Concerns about the worries of those close to you	177 (59.8)	119 (40.2)
Psychological	Anxiety	189 (63.6)	108 (36.4)
Psychological	Learning to feel in control of your situation	189 (63.6)	108 (36.4)
Psychological	Feeling down or depressed	190 (64)	107 (36.0)
Psychological	Feelings of sadness	192 (64.9)	104 (35.1)
Psychological	Worry that the results of treatment are beyond your control	196 (66.2)	100 (33.8)
Psychological	Concerns about the ability of those close to you to cope with caring for you	198 (66.9)	98 (33.1)
Psychological	Keeping a positive outlook	214 (72.1)	83 (27.9)
Psychological	Feelings about death and dying	216 (73)	80 (27.0)
Psychological	Feeling bored and/or useless	216 (73)	78 (26.4)
Sexuality	Changes in sexual feelings	230 (85.8)	38 (14.2)
Sexuality	To be given information about sexual relationships	246 (91.1)	24 (8.9)
Sexuality	Changes in your sexual relationships	256 (94.8)	14 (5.2)
Patient care	Waiting a long time to see your physician	201 (68.1)	94 (31.9)
Patient care	Reassurance by medical staff that the way you feel is normal	203 (69)	91 (31.0)
Patient care	Waiting a long time for clinic appointments	210 (70.9)	86 (29.1)
Patient care	Hospital staff attending promptly to your physical needs	215 (73.1)	79 (26.9)
Patient care	Hospital staff acknowledging and showing sensitivity to your feelings and emotional needs	221 (74.9)	74 (25.1)
Patient care	More choice about which cancer specialists you see	233 (79)	62 (21.0)
Patient care	More fully protected rights for privacy when you’re at the hospital	267 (90.8)	27 (9.2)
Health system	To have one member of hospital staff with whom you can talk to about all aspects of your condition, treatment and follow-up	183 (61.8)	113 (38.2)
Health system	To be given written information about the important aspects of your care	193 (65.2)	103 (34.8)
Health system	To be informed about things you can do to help yourself to get well	192 (65.5)	101 (34.5)
Health system	The opportunity to talk to someone who understands and has been through a similar experience	194 (65.5)	102 (34.5)
Health system	To be informed about cancer which is under control or diminishing (that is, remission)	194 (66.4)	98 (33.6)
Health system	To be given information (written, diagrams, drawings) about aspects of managing your illness and side-effects at home	203 (68.6)	93 (31.4)
Health system	To have access to professional counselling (e.g. psychologist, social worker, counsellor, nurse specialist) if you, family or friends need it	204 (68.9)	92 (31.1)
Health system	To be treated like a person, not just another case	202 (70.4)	85 (29.6)
Health system	To be informed about your test results as soon as feasible	209 (70.6)	87 (29.4)
Health system	To be given explanations of those tests for which you would like explanations	217 (73.6)	78 (26.4)
Health system	To be treated in a hospital or clinic that is as physically pleasant as possible	218 (73.6)	78 (26.4)
Health system	To be given explanations of those tests for which you would like explanations	220 (74.6)	75 (25.4)
Health system	To be informed about support groups in your area	217 (75.1)	72 (24.9)
Financial	Money to cover living expenses	147 (45.2)	178 (54.8)
Financial	Money to treat other illnesses	206 (70.1)	88 (29.9)
Financial	Money to treat side effects of cancer and its treatment	218 (73.9)	77 (26.1)
Reproductive	Need consultation about the reproductive ability	226 (77.1)	67 (22.9)

### Predictors of unmet supportive care needs

Using multivariate generalized linear regression of mean scores, the female gender was independently associated with higher physical (Adj. P = 0.013), psychological (Adj. P = 0.013), patient care and support (Adj. P = 0.001), health system and information (Adj. P = 0.007), and overall need scores (Adj. P < 0.001). Having a comorbid chronic disease was independently associated with higher financial (Adj. P = 0.021), reproductive (Adj. P = 0.040), and overall need scores (Adj. P = 0.011). An educational level of 12 years or less was a significant predictor of higher healthcare system and information need scores (Adj. P = 0.048), while an educational level of > 12 years was a significant predictor for higher overall needs score (Adj. P = 0.038). Being single was independently associated with higher psychological need scores (Adj. P = 0.040). For patients diagnosed with brain tumor malignancies, there was statistically significant and independent correlation with elevated psychological needs score (Adj. P = 0.034). Having socioeconomic challenges was independently associated with higher financial (Adj. P < 0.001) need scores. Being aged 22 or older at the time of diagnosis was a predictor of more healthcare system and information needs (Adj. P = 0.051). The overall QoL score was a significant predictor of more financial needs score (Adj. P = 0.039).

Bivariate and multivariate associations between all independent variables and need domains are presented in Tables 3 and 4.

**Table 3:** Bivariate & Multivariate associations between selected participant characteristics and supportive care need mean scores.

Characteristic/level	Physical			Psychological			Sexuality			Pt care & support		
	Mean score	P	Adj.P	Mean score	P	Adj.P	Mean score	P	Adj.P	Mean score	P	Adj.P
Stage												
Early	27.4	0.380	0.485	30.43	0.647	0.543	2.17	0.411	0.591	30.2	0.253	0.842
Late	20.2			27.08			4.71			22.1		
Gender												
Male	18.8	< 0.001	0.013	24.38	< 0.001	0.013	6.31	0.122	0.323	21.3	< 0.001	0.001
Female	33.9			38.11			10.24			37.7		
Comorbidities												
No	22.1	0.003	0.155	26.89	0.001	0.140	6.76	0.180	0.227	27.5	0.687	0.663
Yes	33.7			38.73			10.55			29.0		
Monthly income												
None	30.9	0.009	0.108	34.39	0.003	0.203	6.70	1.00	0.989	35.3	0.003	0.339
≤ 500 JD	26.9			32.25			7.87			29.1		
> 500 JD	13.4			18.39			8.01			17.4		
Education level												
< 12 years	28.4	0.340	0.238	28.65	0.718	0.347	9.69	0.427	0.820	19.2	0.008	0.177
> 12 years	24.2			30.14			7.21			29.8		
Employment												
Yes	24.5	0.826	0.502	28.97	0.670	0.687	9.31	0.317	0.886	27.3	0.791	0.210
No	25.22			30.38			6.77			28.2		
Governorates												
Amman	24.3	0.662	0.576	29.54	0.805	0.958	8.28	0.535	0.490	27.8	0.964	0.915
Others	25.8			30.34			6.75			28.0		
Marital status												
Not-married	24.5	0.545	0.088	30.02	0.843	0.040	6.26	0.013	0.281	27.3	0.430	0.583
Married	27.1			29.21			14.06			30.5		
Cancer type												
Hematological	22.8	0.552	0.381	27.41	0.057	0.034	6.99	1.00	0.430	27.0	1.00	0.116
Solid tumor	27.9			31.51			9.29			28.5		
Brain cancer	31.9			42.34			8.68			32.1		
Socioeconomic challenges												
Yes	30.0	0.007	0.510	35.3	0.002	0.813	9.1	0.321	0.363	28.4	0.770	0.692
No	20.9			25.5			6.6			27.5		
Time since diagnosis												
≤ 5 years	10.8	0.006	0.146	16.9	0.003	0.232	5.7	1.00	0.614	22.0	0.798	0.152
6–10 years	29.3			35.9			6.5			29.8		
> 10 years	25.7			29.1			9.2			28.1		
Age at collection												
≤ 22	20.9	0.018	0.729	27.5	0.883	0.362	6.1	0.004	0.620	26.2	0.037	0.296
> 22	30.2			32.9			9.8			30.0		
Mean QoL												
Mean score	24.9	0.048	0.179	29.9	0.001	0.709	7.7	0.041	0.129	27.9	0.506	0.102

**Table 4:** Contd. bivariate & multivariate associations between selected participant characteristics and supportive care need mean scores.

Characteristic/level	H. system & information			Financial			Reproductive			Overall domain		
	Mean score	P	Adj.P	Mean score	P	Adj.P	Mean score	P	Adj.P	Mean score	P	Adj.P
Stage												
Early	28.8	0.277	0.682	17.6	0.686	0.866	14.4	0.101	0.175	23.29	0.207	0.990
Late	21.2			20.7			2.1			17.23		
Gender												
Male	21.9	< 0.001	0.007	24.9	< 0.001	0.947	11.4	< 0.001	0.088	18.4	< 0.001	< 0.001
Female	42.9			38.6			32.3			34.1		
Comorbidities												
No	28.2	0.137	0.270	27.1	0.001	0.021	18.7	0.391	0.040	22.9	0.003	0.011
Yes	34.2			41.0			22.9			30.8		
Monthly income												
None	31.2	0.138	0.706	25.6	0.006	0.880	22.6	0.885	0.255	26.6	0.069	0.430
≤ 500 JD	32.1			35.4			17.2			26.3		
> 500 JD	21.1			19.3			25.0			18.5		
Education level												
< 12 years	24.1	0.136	0.048	45.3	0.001	0.156	17.8	0.676	0.582	25.2	0.877	0.038
> 12 years	30.9			27.3			20.1			24.7		
Employment												
Yes	28.1	0.498	0.144	27.3	0.216	0.731	23.8	0.155	0.978	24.96	0.919	0.321
No	30.6			32.1			17.5			24.7		
Governorates												
Amman	29.3	0.837	0.790	28.8	0.291	0.280	18.6	0.516	0.897	24.31	0.594	0.576
Others	30.1			32.8			21.4			25.55		
Marital status												
Not-married	28.7	0.248	0.651	29.8	0.465	0.174	15.0	< 0.001	0.592	23.65	0.029	0.152
Married	34.1			33.3			41.0			30.12		
Cancer type												
Hematological	27.1	0.198	0.245	28.2	0.291	0.398	18.39	1.00	0.648	22.85	0.138	0.123
Solid tumor	32.8			32.0			23.96			27.53		
Brain cancer	40.2			42.0			17.86			30.99		
Socioeconomic challenges												
Yes	33.7	0.037	0.083	41.0	< 0.001	< 0.001	24.2	0.058	0.653	29.59	< 0.001	0.394
No	26.36			21.9			16.1			20.97		
Time since diagnosis												
≤ 5 years	18.3	0.048	0.123	22.8	0.144	0.174	5.5	0.066	0.246	14.69	0.006	0.075
6–10 years	34.1			27.4			20.6			26.74		
> 10 years	29.5			34.9			23.1			26.19		
Age at collection												
≤ 22	27.1	0.008	0.051	30.8	0.734	0.225	13.4	< 0.0001	0.759	22.21	0.01	0.322
> 22	32.8			29.9			28.0			28.13		
Mean QoL												
Mean score	29.7	0.856	0.366	30.4	< 0.001	0.039	19.7	0.165	0.752	24.80	0.003	0.579

### Association between participant characteristics and mean quality of life score

Survivors with socioeconomic challenges reported significantly lower QoL scores, compared to survivors without socioeconomic challenges 72.5 (SD = 21.2) vs. 60.6 (SD = 23.9); P ≤0.001. Additionally, having comorbid chronic disease(s) was significantly associated with a lower mean QoL score compared to patients free of comorbid chronic disease(s) 60.6 (SD = 23.3) vs. 69.3(SD = 22.8); P = 0.006. Survivors with higher educational levels reported a significantly higher mean QoL score compared to survivors with lower educational levels 68.5 (SD = 22.7) vs. 61.3 (SD = 24.6); P = 0.042. Similarly, the type of cancer was significantly associated with the mean QoL score (P = 0.003), and the highest score was reported by survivors of solid tumors 71.6 (SD = 24.2), Table 5.

**Table 5:** Univariate associations between participant characteristics and mean quality of life score.

Characteristics	Levels	Mean QoL score ± STD	P. value
Gender	Male	67.1 ± 23.9	0.976
	Female	67.2 ± 22.0	
Current age	≤ 22 years	68.2 ± 23.2	0.403
	> 22 years	65.9 ± 23.1	
Living situation	Alone	75.0 ± 25.3	0.164
	With a partner	72.2 ± 23.1	
	With parents	66.0 ± 23.0	
Socioeconomic challenges	Yes	60.6 ± 23.9	< 0.001
	No	72.5 ± 21.2	
Monthly income	None	70.1 ± 25.8	0.276
	≤ 500 JD	65.5 ± 23.4	
	> 500 JD	69.9 ± 19.1	
Marital status	Not married	66.7 ± 23.4	0.486
	Married	69.2 ± 22.3	
Employment	Yes	67.9 ± 24.0	0.675
	No	66.8 ± 22.7	
Level of education	≤ 12 years	61.3 ± 24.6	0.042
	> 12 years	68.5 ± 22.7	
Governorate	Amman	67.5 ± 21.9	0.785
	Others	66.7 ± 25.0	
Type of cancer	Hematological	67.4 ± 22.3	0.003
	Solid tumor	71.6 ± 24.2	
	Brain cancer	54.2 ± 22.0	
Cancer stage	Early stage	69.1 ± 18.9	0.236
	Late stage	62.7 ± 20.0	
Time since diagnosis	< 5 years	62.2 ± 20.4	0.282
	5–10 years	69.0 ± 22.2	
	> 5 years	67.2 ± 24.5	
Comorbidities	None	69.3 ± 22.8	0.006
	Yes	60.6 ± 23.3	

### Qualitative data from cancer survivors

**Figure 2:**
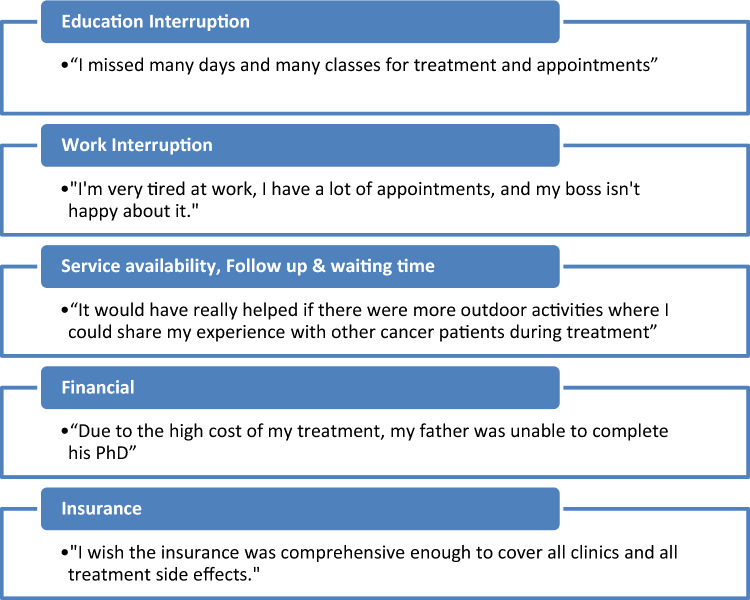
Needs identified in qualitative data.

Other unmet needs were reported by cancer survivors using open-ended questions at the end of the survey. These unmet needs and barriers to survivorship care were identified as a result of content analysis. Survivors reported missing significant amounts of school time due to their treatment schedules, which impacted their education. The need to attend numerous appointments left survivors feeling exhausted and their work was negatively impacted, causing dissatisfaction among both survivors and their employers. There was a desire for more outdoor activities or opportunities to connect with other cancer patients during treatment, indicating a need for improved service availability and patient support. Survivors also expressed concerns about the long wait times for clinic services. Financial burdens and limitations on insurance coverage were also frequently reported by survivors. Themes derived from content analysis with selected illustrative quotes are presented in Fig. 2.

## Discussion

Cancer survivorship is an integral component of the cancer care continuum that begins with diagnosis and continues throughout life. Providing the best possible survivorship care can help patients achieve better health outcomes while living with and after a cancer diagnosis. We started a multi-phase project to explore unmet supportive care needs among cancer survivors. We previously reported needs assessment of adult cancer survivors and predictors of their needs and association with QoL^18^. This current study focuses on adult survivors of childhood cancer which was carried out on a sample at King Hussein Cancer Center. To our best knowledge, this is the first study to report a broad array of unmet supportive care needs among adults who survived childhood cancer in Jordan. Unmet supportive care needs spanned several domains compared with the previously studied adult cancer survivors.

In this population of cancer survivors, female survivors reported higher levels of unmet needs across several domains, including physical, psychological, patient care and support, health system and information, and overall needs. These results are in agreement with those of Cox et al., Burg et al., and Tabrizi et al. which noted a strong relationship between female gender and higher unmet survivorship needs in similar domains^30–32^. Burg et al. uniquely reported unmet financial needs among a Chinese sample of cancer survivors and linked the female gender with higher unmet supportive care needs in patient care and in the impact of cancer on life^33^. Notably, our results differ from those concerning the previously studied population of adult cancer survivors, where gender difference was not a significant predictor of supportive cancer needs, except for reproductive needs^18^. This variation may be attributed to differences in the long-term impact of cancer treatment, psychosocial factors, and life stage challenges specific to surviving childhood cancer. Further exploration is required, which may implicate the need for tailored supportive care strategies.

Recent studies have highlighted the relationship between financial status and unmet supportive care needs among cancer patients^34^ and cancer survivors^35^. The findings derived from the present investigation elucidate no significant relationship between the absence of a regular monthly income and elevated scores in any of the supportive care need domains. On the other hand, outcomes from the multivariate analysis imply that cancer survivors facing socioeconomic challenges tend to report escalated financial needs. This observation is congruent with the existing body of literature, thereby reinforcing previously documented patterns within this research sphere^36,37^ and provides further evidence of the strong association between financial status and different unmet supportive care needs domains.

Our study revealed a significant association between having a comorbid chronic disease and higher need scores in the financial, reproductive, and overall domains. Similar results were reported previously among cancer patients^38^, and patients with other diseases^39^, in addition to cancer survivor populations^40^. Though this is a predictable outcome, it can be explained by the health burden of chronic diseases which pose a need for frequent medical care and follow-up, with its toll of financial and physical implications. Additionally, and in the context of our study’s findings, it is noteworthy to discuss the significant independent association observed between singlehood and psychological need scores among childhood cancer survivors. This suggests that single individuals who have survived childhood cancer may experience unique psychological challenges, likely due to the absence of a partner who could potentially provide emotional sup- port and share the burden of coping with their past cancer experience^33^. The findings from this study reveal a statistically significant and independent correlation between the diagnosis of brain tumor malignancies and a heightened psychological needs score. This result adds to the existing literature by elucidating the unique psychological burden faced by patients diagnosed with brain tumor malignancies^41,42^ and suggesting that these patients may face unique psychological challenges, potentially related to their tumor site and its direct impact on brain functions affecting emotions and cognition, coupled with the uncertainty and severity of the prognosis, and the complexity and invasiveness of treatments.

With regards to the age factor, the present study illuminates a significant relationship between age (22 years and older at the time of data collection) and increased healthcare system and information needs among adult survivors of childhood cancer. Previous reports suggested similar findings in adults and AYA groups^43,44^. This relationship, marginally significant on conventional levels, might be suggestive of a transition point where adult survivors of childhood cancer start to face increased difficulties navigating healthcare systems and require more information about their health and care^45^. This could be attributed to factors such as a shift in healthcare responsibility from parents to themselves, a transition from pediatric to adult care, or increased health complications with age. Future research could benefit from a more detailed exploration of age-related factors among adult survivors of childhood cancer by delineating specific age ranges. This approach could provide deeper insights into the evolving healthcare needs and challenges faced by these survivors at different stages of adulthood, enhancing the development of age-appropriate care strategies.

An educational level of less than or equal to 12 years was a significant predictor of higher financial needs, which is similar to the results of our previously studied adult cancer survivors population^18^. Previous reports from the literature also reported unmet needs to be greatest in those with lower educational status, which could be attributed to lower chances of finding good jobs; therefore, as discussed above financial hardships are expected to be higher^46,47^.

Aligning with prior research, quality of life (QoL) has been identified as a predictive factor for unmet needs in our study. We observed a significant inverse association between QoL and financial needs. While this could suggest that childhood cancer survivors with higher QoL have lesser financial support requirements, it is also possible that the direction of this association may be reversed. In other words, lower financial needs might contribute to a higher QoL. This possibility aligns with the nature of correlational studies where the direction of causality is not always clear. This nuanced interpretation is supported by recent empirical findings in this research domain^48–50^, which also explore the complex interplay between QoL and various needs of cancer survivors.

Unmet needs and barriers to survivorship care that were identified as a result of content analysis of open-ended questions were mostly related to school and education, work interruptions, as well as service availability, and the need to reduce waiting times at the clinics. Financial burdens, follow-up on the side effects of cancer treatment, and limitations on insurance coverage were also frequently reported by survivors. All of these needs had previously been discussed in many qualitative studies in the literature^17,51–53^. Work and education interruptions were the top two unmet needs reported by survivors, which are known to be related to the late effects of cancer that affect both survivors’ educational and occupational status^54^.

The main strength of this study lies in the use of the validated SCNS-SF instrument for assessing the needs of cancer survivors, which was developed in an oncology population and addressed a wide range of needs. In addition, open-ended questions were included asking survivors about other needs to avoid overlooking unidentified areas in the instrument. Moreover, this study is part of a series of studies designed to increase our understanding of unmet supportive care needs among Jordanian-Arab cancer survivors, and this paper at hand reports, and for the first time, the supportive care needs of a unique population of Arab cancer survivors who experienced various cancer types during childhood.

Self-reporting of unmet needs may be considered a limitation in this study because some childhood cancer survivors may have been more reluctant or unable to participate due to long-term side effects, for example. This could have resulted in under or over-reporting of unmet needs. Furthermore, the research was carried out in a tertiary-level cancer center, where patients have easier access to healthcare providers such as oncologists, nurse care coordinators, and psychologists, which may reflect a lower level of unmet needs compared to other healthcare settings. Moreover, we acknowledge that the inability to contact 41% of potential participants due to disconnected telephone numbers represents a significant limitation of our study. This high non-contact rate may have introduced selection bias, as it was not feasible to compare the demographic and clinical characteristics of these individuals with those who participated. Future studies should consider employing diverse methods of participant contact, such as postal mail, and strive for access to comprehensive registry data to better understand and mitigate potential biases.

The findings of our study may not be applicable to countries with different healthcare systems, but they can serve as a model for other neighboring Arab countries due to cultural and linguistic similarities. This is the first study in Jordan and among very few in the region that focus on this unique population of adult cancer survivors. It also adds more evidence and builds on current literature that aims to extend our understanding of unmet supportive care needs among Jordanian-Arab cancer survivors, and highlights opportunities for interventions and improved cancer care.

## Conclusion

We report the presence of several unmet needs in this population of cancer survivors spanning many domains. In this unique adult cancer survivor group, female gender, financial factors, socioeconomic challenges, level of education, patients diagnosed with brain tumors, and lower quality of life levels were associated with unmet supportive care needs. Results from this study confirm the need for more tailored studies assessing different populations of cancer survivors and avoiding the one size fits all model when it comes to survivorship care. The noted differences between the reported needs of adult survivors and this population of adult survivors of childhood cancer suggest that cancer experiences during childhood significantly influence supportive care needs during adulthood. Assessed needs were highest in the financial domain, including managing adverse effects and comorbidities of cancer treatment. Incorporating information on how to mitigate financial issues into cancer survivorship plans gives an opportunity to address a frequently occurring patient need. Assisting patients in locating convenient solutions to lessen the financial burden and cost of treatment can have a profound impact on survivors care and outcome. Understanding these findings highlights opportunities for interventions to address gaps in care.

## Data Availability

The data that support the findings of this study are available upon reasonable request from the corresponding author.
